# Adolescent psychiatric outpatients and their caregivers: Comparing the Strengths and Difficulties Questionnaire

**DOI:** 10.4102/sajpsychiatry.v26.i0.1394

**Published:** 2020-11-09

**Authors:** Yashna Mohangi, Thulisile G. Magagula, Deborah van der Westhuizen

**Affiliations:** 1 School of Medicine, Faculty of Health Sciences, University of Pretoria, Pretoria, South Africa; 2 Department of Psychiatry, Weskoppies Hospital, Pretoria, South Africa

**Keywords:** adolescent, Strengths and Difficulties Questionnaire, caregiver, psychiatry, outpatients

## Abstract

**Background:**

The use of the SDQs as a screening tool to monitor new or ongoing problems in adolescent psychiatric outpatients is needed to improve clinical outcomes. Discrepancies between parent and adolescent reports on the Strengths and Difficulties Questionnaire (SDQ), reflects the degree of emotional and behavioural symptoms. This may affect their ability to work together to reach therapeutic goals.

**Aim:**

The level of SDQ (dis)agreements between adolescent-parental self-reports in adolescent psychiatric outpatients was examined.

**Setting:**

Weskoppies Child and Adolescent outpatients.

**Methods:**

This two-group cross-sectional comparative study obtained SDQ responses from 74 psychiatrically diagnosed adolescents and their parents (148 completed SDQ questionnaires). Adolescent outpatients aged between 11 and 18 years following up at the outpatients between July 2017 and November 2017 were included. Adolescent and parent rating scores were compared using a paired sample t-test, and patterns of agreement were measured by using Pearson’s correlation coefficient and Cohen’s kappa.

**Results:**

Parents reported more difficulties than adolescents, although differences were non-significant (*p* > 0.58). Caregivers and adolescents agreed on the conduct domain and on emotional symptoms (0.21 ≤ kappa ≤ 0.40, *p* < 0.05). Caregivers and adolescents agreed on the presentation of internalising and externalising disorders (*R* = 0.48, *p* < 0.001).

**Conclusions:**

The SDQ confirmed fair agreement between parents and adolescents. Parental perceptions of adolescent behavioural difficulties could influence parent– adolescent relations and communication. Using the SDQ as a screening tool in South Africa, requires further validation for it to be integrated as part of a multi-informant best-practice approach.

## Introduction

Child and adolescent psychiatric disorders usually result in severe distress and social impairment. Global epidemiological data indicate that up to 20% of children and adolescents suffer from impairing mental illness, with suicide being the third leading cause of death amongst adolescents. Up to 50% of all adult mental disorders show an onset during the adolescent years.^1^ These statistics indicate the importance of timeously identifying both new and ongoing emotional and behavioural difficulties experienced by children.

Undoubtedly, parents, or the primary caregivers of the child, are often the most frequent observers of these behaviours,^2^ and historically, only their observations of the child’s symptoms were included in the assessment process. However, children are still the most accurate raters of their own experiences, especially of emotional symptoms.^2^ Given the value of both self- and caregivers’ observations, psychological problems in adolescents should ideally be evaluated by using a multi-informant approach.^3^ One way to gather information relevant to a specific setting is by using screening questionnaires. Using the Strengths and Disabilities Questionnaire (SDQ) as a screening questionnaire has the advantage of being less time consuming and easier to administer, becoming increasingly favourable in public mental health services.^4,5^ Screening questionnaires are particularly useful in busy outpatient settings, where there is often not enough time or expertise to conduct clinical interviews.^4,5^ Questionnaires that are completed by both the parent and the child can be used in addition to clinical interviews, or they can be used as screening tools during the assessment process.^3^ Questionnaires completed by adolescents and caregivers allow the clinician to compare assessments and gain an impression of current psychopathological symptoms.^5^

The clinical outcomes of psychiatric treatment require continuous monitoring of psychopathological symptoms. During treatment, changes that are evaluated from multiple perspectives are known to show discrepancies in the perception of children’s mental health. These discrepancies could lead to different interpretations of treatment outcomes.^6^ The discrepancies between youth self-reports and parental reports should be investigated to rectify different perceptions and problems expressed by adolescents and caregivers.^7^ One such area of concern is when adolescents and caregivers report contradicting information on emotions. Different perceptions of emotions may indicate that caregivers are not sensitive to emotional disturbances in their child or adolescent.^2,8^ Different reports from caregivers and adolescents may also indicate a possible relapse in children who have been receiving treatment for mental health problems. In contrast, reports that are in strong agreement may indicate that the caregiver is in tune with the child’s needs, and that the patient is more likely to be well controlled as an outpatient.

One such questionnaire that is used to simultaneously evaluate both the adolescent patient and the caregiver is the SDQ. The SDQ is a short behavioural screening instrument developed by Goodman, which can be administered to caregivers, including mothers, fathers, teachers, friends and others as informant reports, and to children and adolescents as self-reports.^4,8,9^ The SDQ is comprised of 25 psychological attributes that contain positive and negative behavioural traits.^4^ The five subscales each have five points that cover emotional, conduct, hyperactivity and peer problems, and prosocial behaviour. The extended version of the SDQ includes a brief impact supplement. The SDQ has been used in South Africa and is available in multiple languages. In psychiatric clinical samples, the findings of the SDQ correlate well with clinical diagnoses.^9^ In fact, a study comparing the SDQ with the Child Behaviour Checklist (CBCL), where the latter contains 118 items on psychopathology alone, found the SDQ to be superior at detecting inattention and hyperactivity and just as good at detecting internalising and externalising problems.^10^ Internalising problems include diagnoses of mood and anxiety disorders, whereas externalising disorders include behavioural problems such as conduct disorders and attention-deficit hyperactivity disorder (ADHD).

Despite the ready availability of the SDQ, few studies have used the tool in South African clinical settings. A recent review on the use of the SDQ in Africa^11^ found that it was a useful screening tool to identify adolescents at risk of mental health problems in a quarter of African countries.^11^ However, in Africa, the SDQ has not been psychometrically evaluated as a tool when adequately following the guidelines.^11^ In this study, we apply the SDQ in a clinical setting and compare the SDQ reports of treated adolescents and their caregivers in an outpatient psychiatric unit in South Africa. Although many factors could affect adolescent–parent (dis)agreements, such as relations and communication characteristics, research has suggested that this may indicate treatment failure or new psychiatric problems. Reports that agree between adolescents and caregivers may imply stability and treatment success. The meaning of parent–adolescent (dis)agreement should be seen in the context of relationships and/or family social status and should be integrated in the overall understanding of the adolescent’s situation.^8^

## Methods

In this quantitative, two-group cross-sectional comparative study, we compared SDQs with a brief impact supplement, completed by adolescents and caregivers. We conducted this study between 01 July 2017 and 30 November 2017. The adolescents being treated at the Weskoppies Child and Adolescent Unit outpatient department were further categorised into patients with externalising disorders, such as conduct disorder and ADHD, or patients with internalising disorders, such as mood and anxiety disorders. The outpatient department provides tertiary-level mental health services to children and adolescents in the Pretoria area.

The participants are comprised of 74 adolescent–caregiver pairs. We used the statistician recommended sample size and used a convenience sample, which was restricted to the particular age group and level of literacy. The adolescents were previously diagnosed and treated outpatients, who were accompanied by a caregiver, could speak English and are literate. Adolescents were only considered and included if they were able to assent and the caregiver was able to give consent for completing the SDQ. Each caregiver and adolescent completed his or her relevant SDQ with assistance from the researchers.

## Objective

The aim of this study was to evaluate the agreement and disagreement between adolescents’ self-report and caregivers’ report on the various SDQ domains; overall (dis)agreement between caregiver reports and adolescent self-reports regarding either internalising or externalising disorders will also be reported.

## Statistical methods and data analysis

The level of agreement between adolescent’s self-reports and caregiver’s reports was assessed by using Cohen’s kappa statistic for the SDQ domains. We used Hotelling’s paired T2 test to determine if there was a difference between caregiver- and adolescent-rating scores; differences were deemed to be significant if *P* < 0.05. To see if adolescent and parent total difficulty scores were related, we used a Pearson’s correlation coefficient. Cohen’s kappa measure of agreement < 0 indicates no agreement; 0.001–0.20 a weak agreement; 0.21–0.40 a fair agreement; 0.41–0.60 a moderate agreement; 0.61–0.80 a substantial agreement; and 0.81–1.00 an almost perfect agreement. The adolescents were further divided into internalising and externalising groups based on their previous clinical diagnoses as obtained from the treating team. We used a bivariate Pearson correlation test to evaluate agreement in each of the groups.

## Ethics consideration

Participants had to be literate in English, and able to give written informed consent and assent. To ensure confidentiality, no patients’ names were written on the questionnaires. The protocol was approved by the University of Pretoria, Faculty of Health Sciences and Research Ethics Committee (reference number: 219/2017).

## Results

In total, 74 adolescent–caregiver pairs completed the SDQ. Of these 74, 54% were girls and 46% were boys. Girls reported slightly more difficulties overall (22.08, 55.19%) compared with boys (20.26, 50.66%) (Table 1). In contrast, caregivers reported slightly higher difficulty scores for boys (21.65 ± 5.79, 54.12%) compared with girls (21.60 ± 4.75, 54%) (Table 1). There was no significant difference between self-report scores and caregiver scores, between boys and girls, for all the different subscales of the SDQ, namely ‘emotional symptoms, conduct problems, hyperactivity symptoms, peer problems and total difficulties’ (Table 1).

**TABLE 1:** The Strengths and Disabilities Questionnaire scores for boys and girls, and their parents.

Subscale of SDQ	Male report				Female report			
	Mean ± SD		*p*	Correlation	Mean ± SD		*p*	Correlation
	Parent report	Child report			Parent report	Child report		
Emotional symptoms	5.21 ± 2.95	4.94 ± 2.36	0.61	0.37	4.53 ± 2.73	4.83 ± 2.61	0.58	0.21
Conduct problems	4.06 ± 1.63	3.47 ± 1.78	0.17	0.13	5.03 ± 2.14	5.08 ± 2.25	0.89	0.44
Hyperactivity symptoms	4.85 ± 1.84	5.12 ± 2.10	0.57	0.08	5.13 ± 1.47	5.85 ± 1.58	0.16	0.28
Peer problems	5.53 ± 1.52	5.74 ± 1.90	0.57	0.25	4.93 ± 1.58	5.33 ± 1.69	0.22	0.22
Total difficulties	21.65 ± 5.79	20.26 ± 5.25	0.19	0.40	21.60 ± 4.75	22.08 ± 0.39	0.60	0.38
Prosocial	7.68 ± 1.92	8.06 ± 2.56	0.44	0.21	6.63 ± 1.98	7.48 ± 2.12	0.06	0.12

SDQ, Strengths and Disabilities Questionnaire; SD, standard deviation.

Note: Boys and girls were receiving treatment at the Weskoppies Child and Adolescent Unit outpatient department (2017).

**FIGURE 1:**
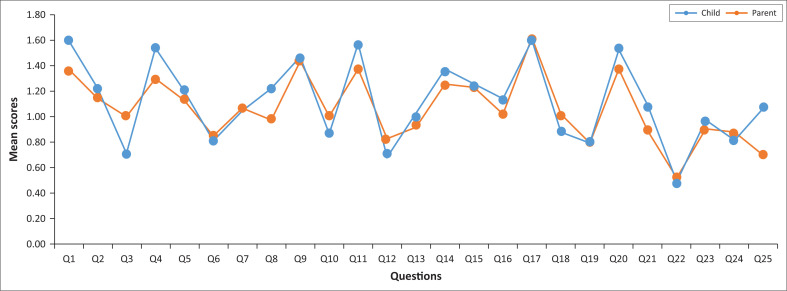
Comparison of the mean scores per question of the Strengths and Disabilities Questionnaire completed by adolescent–caregiver pairs; adolescents were receiving treatment at the Weskoppies Child and Adolescent Unit outpatient department (2017).

For the whole group (Table 2, Figure 1), the total difficulty scores for adolescents were lower than those of their caregivers, but not statistically significant (*P* = 0.58). Adolescent and caregiver total difficulty scores were positively but weakly correlated (*r* = 0.38). The only domain where adolescents’ and caregivers’ scores were significantly correlated was the domain conduct problems (*r* = 0.40, *p* = 0.05). The largest difference in adolescent–caregiver scores was observed in mean prosocial rating scores (Table 2). Adolescent’s and caregiver’s scores did not differ significantly in any of the domains (Table 2).

**TABLE 2:** The Strengths and Disabilities Questionnaire scores for all adolescents, and their parents.

Subscale of SDQ	Adolescent Mean ± SD	Caregiver Mean ± SD	*p*	Correlation
Emotional symptoms	4.84 ± 2.83	4.88 ± 2.48	0.91	0.28
Conduct problems	4.58 ± 1.97	4.34 ± 2.19	0.36	0.40
Hyperactivity symptoms	5.00 ± 1.65	5.51 ± 1.86	0.54	0.18
Peer problems	5.20 ± 1.57	5.51 ± 1.79	0.20	0.25
Total difficulties	21.62 ± 5.22	21.24 ± 5.36	0.58	0.38
Prosocial	7.11 ± 2.01	7.74 ± 2.33	0.05	0.19

SDQ, Strengths and Disabilities Questionnaire; SD, standard deviation.

Note: Adolescents were receiving treatment at the Weskoppies Child and Adolescent Unit outpatient department (2017).

## The conduct domain

There was a fair agreement between adolescent’s and caregiver’s scores for most questions in the conduct domain (Table 2). Adolescents and caregivers did not agree on question 5 (*P* = 0.1754) (Table 2).

## The emotional symptom domain

Adolescent’s and caregiver’s scores on emotional symptoms showed a fair agreement for questions 3, 8 and 24 (0.21 ≤ kappa ≤ 0.40, *P* < 0.05). A weak agreement was observed for questions 13 (kappa = 0.17, *P* < 0.05) and 16 (kappa = 0.02), see Table 4.

**TABLE 3a:** Comparison of conduct domain scores for adolescent–caregiver pairs.

Variable	Q5			Q7			Q12			Q18			Q22		
	NT	ST	CT	NT	ST	CT	NT	ST	CT	NT	ST	CT	NT	ST	CT
NT (disagree)	7	4	8	7	7	4	25	8	6	16	8	6	41	6	3
ST (agree somewhat)	3	10	7	4	22	8	7	4	7	4	9	9	6	2	5
CT (agree totally)	9	12	14	7	4	11	2	6	9	4	8	10	2	3	6
**Total**	**19**	**26**	**29**	**18**	**33**	**23**	**34**	**18**	**22**	**24**	**25**	**25**	**49**	**11**	**14**

NT, not true; ST, somewhat true; CT, certainly true (three available responses per the Strengths and Disabilities Questionnaire).

Note: Q relates to question numbers as per the Strengths and Disabilities Questionnaire.

**TABLE 3b:** The Cohen’s kappa measure of agreement.

Variable	Q5	Q7	Q12	Q18	Q22
Kappa value	0.1112	0.2106	0.286	0.3221	0.2283
Approx. significance	0.1754	0.0098	0.0006	0.0002	0.006

Note: Q relates to question numbers as per the Strengths and Disabilities Questionnaire.

**TABLE 4a:** Comparison of emotional symptoms domain scores for adolescent–caregiver pairs.

Variable	Q5			Q7			Q12			Q18			Q22		
	NT	ST	CT	NT	ST	CT	NT	ST	CT	NT	ST	CT	NT	ST	CT
NT (disagree)	23	4	12	7	6	3	13	10	3	4	7	9	20	13	4
ST (agree somewhat)	1	8	9	6	15	5	3	12	7	11	9	4	3	6	4
CT (agree totally)	4	5	8	9	10	13	7	11	8	9	8	13	6	6	12
**Total**	**28**	**17**	**29**	**22**	**31**	**21**	**23**	**33**	**18**	**24**	**24**	**26**	**29**	**25**	**20**

NT, not true; ST, somewhat true; CT, certainly true (three available responses per the Strengths and Disabilities Questionnaire).

**TABLE 4b:** Cohen’s kappa measure of agreement.

Variable	Q5	Q7	Q12	Q18	Q22
Kappa value	0.27755	0.20845	0.17644	0.02418	0.25959
Approx. significance	0.0005	0.00945	0.02682	0.76797	0.00113

## Internalising and externalising disorders

Of the 74 adolescents, 58 adolescents had internalising disorders and 47 adolescents had externalising disorders. Thirty-two adolescents were diagnosed with a combination of internalising and externalising disorders. One adolescent did not have a diagnosis (party number 28). In adolescents with internalising disorders, adolescents had mean SDQ scores of 21.78 (54.4%) and caregivers had mean SDQ scores of 21.95 (54.9%). The total difficulties scores for adolescents with internalising disorders and their caregivers were positively, but weakly, correlated (*r* = 0.328, *P* = 0.12). Adolescents with externalising disorders had mean SDQ scores of 21.30 (53.2%), and their caregivers had scores of 22.00 (55%). The total difficulties scores for adolescents with externalising disorders and their caregivers were positively correlated (*r* = 0.481, *P* = 0.001) (Table 5).

**TABLE 5:** The Strengths and Difficulties Questionnaire (SDQ) scores for all adolescents, and their parents.

Variable	Internalising group		Externalising group	
	Mean	%	Mean	%
Parent	21.95	54.87	22.0	55.00
Child	21.78	54.44	21.3	53.24

Note: Adolescents are grouped into those with internalising or externalising disorders.

Total difficulties scores: Internalising group, *r* = 0.328, *p* = 0.12; Externalising group, *r* = 0.481, *p* = 0.001.

Of the 74 adolescent–caregiver pairs, 43 (58.1%) agreed on the adolescent’s state since their coming to the clinic. Most (44.1%) reported being ‘much better’ (44.1%), and these were caregivers of male adolescents. More than 80% of both adolescents and caregivers reported that their problems were better, either a bit better or much better, or both (see Table 6).

**TABLE 6:** Impact scale for all adolescents, and their parents.

Variable	Muchworse		A bitworse		Same		A bitbetter		Muchbetter		Grandtotal	
	*n*	%	*n*	%	*n*	%	*n*	%	*n*	%	*n*	%
Female child	1	2.5	1	2.5	4	10	17	42.5	17	42.5	40	100
Male child	1	2.9	2	5.9	3	8.8	15	44.1	13	38.2	34	100
**Total child**	**2**	**2.7**	**3**	**4.1**	**7**	**9.5**	**32**	**43.2**	**30**	**40.5**	**74**	**100**
Female parent	1	2.5	2	5.0	2	5.0	19	47.5	16	40	40	100
Male parent	0	0	0	0	8	23.5	11	32.4	15	44.1	34	100
**Total parent**	**1**	**1.4**	**2**	**2.7**	**10**	**13.5**	**30**	**40.5**	**31**	**41.9**	**74**	**100**

Note: Adolescents were receiving treatment at the Weskoppies Child and Adolescent Unit outpatient department (2017).

## Discussion

In this study, we assessed the level of agreement between SDQs completed by adolescent outpatients receiving psychiatric treatment and the SDQs completed by their parents. We did not find any differences between adolescent’s and caregiver’s SDQ scores. Adolescent and caregiver scores for the prosocial domain were correlated, and various aspects in the conduct and emotional symptoms domain showed strong agreement. The strong agreement between adolescent and parent reports may be because of the fact that most of the adolescents were being treated at the clinic, and most of the adolescent–caregiver pairs reported improved mental health.

When we analysed the total difficulty scores according to sex, girls had higher self-reported total difficulty scores compared to with boys (Table 1). Previous studies^3,8,12^ have also found that girls report more problems than boys. This trend has been attributed to the fact that girls are able to gauge and recognise their difficulties more than boys, who may under-report their symptoms because of denial or higher levels of symptom tolerance.^2,8^ Boys may also be more restrained when discussing their emotions and their feelings.^13^ Girls may be more sensitive to the effect that these difficulties have on their social lives, whereas boys are more aware of problems that influence their interaction with peers as assessed by the ‘peer problems scale’ on the SDQ. Having friends and maintaining successful friendships are important for an adolescent’s psychological well-being and development, with peer interaction now being recognised as a potential therapeutic instrument.^8,14^ Whilst girls reported higher difficulty scores, the caregivers reported that their boys had more difficulties, which is consistent with previous studies where parents report more problems for boys than girls.^3^ Presumably, parents perceive their boys to have more problems, because boys are prone to higher rates of externalising disorders compared with girls who are usually diagnosed with internalising disorders. Boys tend to exhibit more obvious behaviour problems,^3^ which are more easily observed and reported by parents. Despite these slight differences, most scores reported by adolescent–caregiver pairs were in agreement.

Previous studies have also reported a strong agreement between adolescent–caregiver pairs’ difficulties scores for adolescents who are receiving treatment.^4,15,16,17^ Parent–child agreement in a treated and stabilised group of individuals may suggest that parents are more sensitive to their child, are now more attentive and have been advised on how to manage their behaviour, which may enable them to pick up signs of early relapse. Other studies using community samples have also found that the SDQ scores of older children and their parents are usually in agreement,^18^ and that there is less disagreement between children and their parents, as the children become older.^6,19^ Using the SDQ to screen younger patients may yield different results.

Of particular interest are specific domains of the SDQ that yielded significant agreement between adolescent and caregiver reports. Adolescents and caregivers agreed on most aspects of the conduct domain (Table 3). The high agreement in total conduct scores between adolescents and caregivers could be because of adolescents being more aware of their externalising difficulties, as these difficulties are easily observed. Older children who are being treated are more likely to be aware of previous behaviour problems, may be more cognitively mature and be able to distinguish this behaviour along with their caregivers. The conduct domain includes questions such as ‘I usually do as I am told’, ‘I fight a lot’, ‘I am often accused of lying or cheating’ and ‘I take things that are not mine’. There was least agreement between scores for question five (Table 3). Question 5 assessed temper tantrums, which refers to a child being very angry and in a state of emotional distress to a point that decreases self-awareness, whereby a parent can easily observe an outburst. The poor agreement for question 5 suggests ongoing difficulties with conduct problems despite treatment. Previous studies have reported higher correlations between SDQ scores of caregivers and adolescents who have externalising disorders,^8,20^ similar to our results. Previous studies have also shown that adolescents who are receiving treatment for mental problems are better able to recognise their own conduct disorder than a control group of adolescent patients without medical diagnosis.^21,22^ The high level of agreement observed in our study may indicate greater levels of self-realisation and acknowledgement by the adolescents, and may be an opportunity to apply behaviour modification techniques such as cognitive behaviour therapy (CBT) for future treatment.

The other domain, which showed fair levels of agreement in our study, was the emotional symptoms domain. Our sample was comprised of diagnosed and treated outpatients. Children and adolescents who have been diagnosed with emotional disorders have been found to show vast improvement during outpatient treatment as measured by the SDQ.^23^ The agreement between adolescents and caregivers in the emotional symptoms domain could be as a result of adolescents being more open about their emotions to their parents, who are also more receptive and sensitive to the emotional needs of their child after attending and receiving help from an outpatient clinic. Similar results were reported from China,^13^ where parents and adolescents share close relationships. In close adolescent–caregiver relationships, parents may be more attentive and better understand the emotional problems of their children. The weakest level of agreement related to questions 13 and 16, which asked about feelings of unhappiness and nervousness in new situations, respectively. These questions are very subjective and hint at internalising symptoms, which parents are often unaware of. Various reports^3,8^ support the theory that parents are not able to detect symptoms of internalising disorders as readily as externalising disorders.

In our study, parents of adolescents with externalising disorders reported slightly higher mean difficulty scores than parents of adolescents with internalising disorders. Previous studies have confirmed that caregivers of adolescents with externalising disorders report more observable behaviour than caregivers of children with internalising difficulties.^3,8^ For adolescents with externalising disorders, the adolescent difficulty scores were positively correlated with caregiver scores (Table 5).

Whilst completing the SDQ, we also asked adolescent–caregiver pairs to complete the impact scale, to look at the impact of these difficulties on the lives of parents and adolescents. Most of the adolescent–caregiver pairs agreed that their adolescents were either ‘a bit better’ or ‘much better’. Most adolescent–caregiver pairs agreed that attending an outpatient clinic was helpful, as they observed an overall improvement in their child’s well-being. Special outpatient clinics provide access to child and adolescent psychiatrists, psychologists and social workers.

## Strengths and limitations

Our study compared the SDQ scores reported by 74 adolescents, aged between 11 and 18 years old, and their caregivers in a clinical outpatient group. Even though the SDQ is easy to use, inexpensive and reliable, studies that use the SDQ are very limited in non-Western societies. The SDQ is widely used in many countries in clinical settings as a tool for screening, diagnosing and monitoring psychiatric disorders. The SDQ enables the child to report important information from a subjective perspective. These self-reports form part of multi-informant reports that can be used to form holistic diagnoses and treatment plans. The small sample size is a limitation and may limit the generalisability of our results. The second limitation is that only dyads that were proficient in English were included. We initially planned on having a larger sample size, but were limited by the number of adolescents who were able to understand and self-complete the SDQ, and by the number of adolescents who had a caregiver present.

## Conclusion

In this clinical group of adolescents and their caregivers, the SDQ scores were similar and in agreement with each other, which was in contrast to other studies. Overall, adolescent–caregiver pairs were in agreement for total difficulties, which implies a good treatment progress with an opportunity for further improvement in clinical care. The strongest agreement between adolescent–caregiver pairs was observed in the conduct domain, indicating a greater awareness of observable behaviour problems. Caregivers of adolescents with externalising problems showed an even stronger agreement. Adolescent–caregiver pairs agreed that attending an outpatient psychiatric service has assisted in improving their ongoing difficulties. Whilst it should be acknowledged that the use of the SDQ as a screening tool in South African still needs to be standardised, its use shows value in adolescent mental health.
